# Prospective applications of extracellular vesicle-based therapies in regenerative medicine: implications for the use of dental stem cell-derived extracellular vesicles

**DOI:** 10.3389/fbioe.2023.1278124

**Published:** 2023-10-23

**Authors:** Wenhao Wang, Zinan Xu, Minyi Liu, Mingxiang Cai, Xiangning Liu

**Affiliations:** ^1^ School of Stomatology, Jinan University, Guangzhou, China; ^2^ Center of Stomatology, The First Affiliated Hospital of Jinan University, Guangzhou, China; ^3^ Clinical Research Platform for Interdiscipline, Jinan University, Guangzhou, China

**Keywords:** extracellular vesicles, engineering modification, regenerative medicine, dental stem cells, circulation time, targeting

## Abstract

In the 21st century, research on extracellular vesicles (EVs) has made remarkable advancements. Recently, researchers have uncovered the exceptional biological features of EVs, highlighting their prospective use as therapeutic targets, biomarkers, innovative drug delivery systems, and standalone therapeutic agents. Currently, mesenchymal stem cells stand out as the most potent source of EVs for clinical applications in tissue engineering and regenerative medicine. Owing to their accessibility and capability of undergoing numerous differentiation inductions, dental stem cell-derived EVs (DSC-EVs) offer distinct advantages in the field of tissue regeneration. Nonetheless, it is essential to note that unmodified EVs are currently unsuitable for use in the majority of clinical therapeutic scenarios. Considering the high feasibility of engineering EVs, it is imperative to modify these EVs to facilitate the swift translation of theoretical knowledge into clinical practice. The review succinctly presents the known biotherapeutic effects of odontogenic EVs and the underlying mechanisms. Subsequently, the current state of functional cargo loading for engineered EVs is critically discussed. For enhancing EV targeting and *in vivo* circulation time, the review highlights cutting-edge engineering solutions that may help overcome key obstacles in the clinical application of EV therapeutics. By presenting innovative concepts and strategies, this review aims to pave the way for the adaptation of DSC-EVs in regenerative medicine within clinical settings.

## 1 Introduction

Regenerative medicine aims to restore the functionality of impaired organs or tissues. Different strategies are being explored to achieve this goal. The majority of these approaches focus on the utilization of diverse primary cells, including stem cells and their secretions (Yin et al., 2020; Nagelkerke et al., 2021). Among these approaches, stem cell transplantation stands out as the strategy with the most potential. In particular, mesenchymal stem cells (MSCs) derived from bone marrow have garnered substantial attention due to their clinical significance and extensive exploration (Ghiroldi et al., 2018; Rozier et al., 2018; Tatullo, 2019). Nonetheless, it is crucial to note that the isolation of these adults stems from human bone marrow (hBM) necessitates relatively invasive procedures that are not well accepted by patients (Beyer Nardi and da Silva Meirelles, 2006). Hence, there is a pressing need to explore alternative sources of MSCs that align more seamlessly with clinical practices in future scientific investigations. Recent years have witnessed a growing body of research reporting that dental stem cells (DSCs) are more plastic and proliferative and have better immunomodulatory properties than bone marrow stem cells (BMSCs) (Mantesso and Sharpe, 2009). Numerous studies have observed the potential of dental pulp stem cells (DPSCs) to develop not only into bone, adipose, and cartilage but also into myocytes, hepatocytes, and neurons (Iohara et al., 2006). Given their self-renewal capacity and remarkable plasticity, these cells hold promise for diverse clinical applications in both dentistry and medicine. However, it is important to acknowledge that the clinical utilization of stem cells still confronts numerous challenges. The foremost impediment to the widespread adoption of stem cell therapy, resides in its safety profile, primarily characterized by risks such as pro-inflammation, tumorigenicity, and host rejection (Caplan et al., 2019). In addition, their structural contribution to tissue regeneration is more constrained than initially believed, with only a fraction of the delivered cells successfully integrating into the host and differentiating into the intended cell type (Baraniak and McDevitt, 2010). In contrast, Valadi. et al. elucidated the role of horizontal mRNA transfer through extracellular vesicles (EVs) as a mechanism facilitating the paracrine exchange of genetic information between MSCs and other cells (Valadi et al., 2007). As elucidated in the research conducted by Gnecchi et al., MSCs exert their effect primarily through paracrine mechanisms (Gnecchi et al., 2005; Phan et al., 2018). In the field of regenerative medicine, the utilization of MSCs is primarily dependent on their capacity to produce cytokines and growth factors rather than on their inherent self-renewal and differentiation potential (Pittenger et al., 2015). Notably, EVs derived from MSCs exhibit intricate biosynthetic mechanisms and distinctive structural features. They also possess the regeneration potential inherent to MSCs of specific origin and maintain the ability to migrate their parental cells to particular tissues (Lener et al., 2015; Phinney and Pittenger, 2017). These unique attributes make MSC-derived EVs highly beneficial to the regeneration process.

**FIGURE 1:**
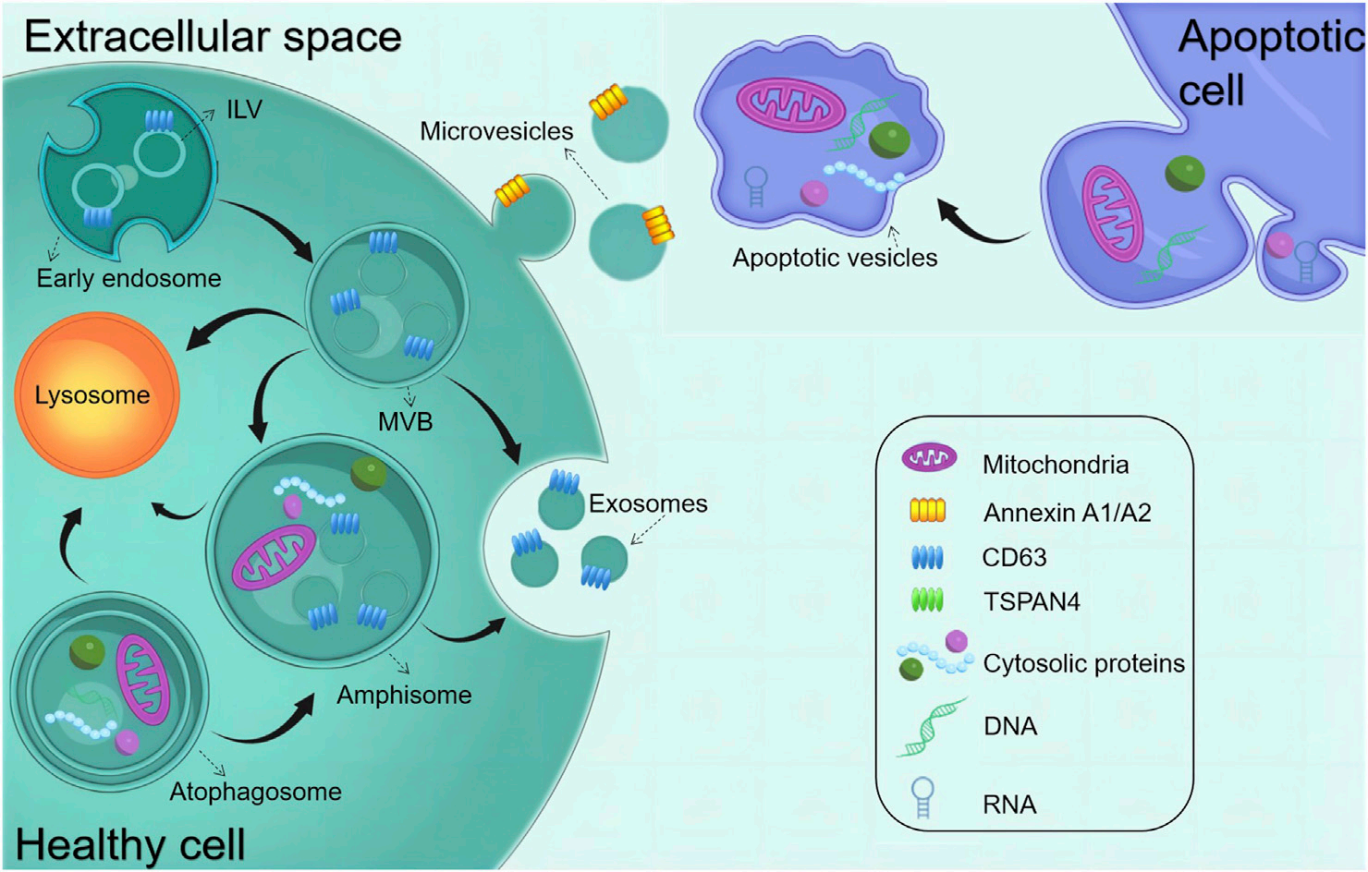
Biogenesis of common EVs. The early endosome matures and starts forming intraluminal vesicles (ILVs) (future exosomes), thereby transforming into multivesicular bodies (MVBs). MVBs can fuse with either autophagosomes or lysosomes to be degraded. MVBs are also transported to the plasma membrane to release exosomes via exocytosis. Amphisomes are formed by the fusion of autophagosomes with MVBs. Exosomes are released by the exocytosis of MVBs and amphisomes. Microvesicles are produced through plasma membrane budding and blebbing. Apoptopodia release apoptotic vesicles during apoptosis.

Almost all tissues and cells secrete EVs and transmit signals and effector molecules across cells via circulating body fluids, making them a promising candidate for medical diagnostics and therapy. EVs comprise a homogeneous lipid bilayer in which substances, including proteins, RNAs, and metabolites, are selectively packaged. Consequently, they assume a distinctive role in facilitating intercellular communication, as highlighted in various studies (Lässer et al., 2018; Phan et al., 2018). EVs released from different sources exert influence over the regulatory processes of the cell cycle by mediating intracellular signaling, including the regulation of physiological activities such as proliferation or differentiation. Serving as pivotal molecules in intercellular communication, EVs play a major role in mediating the exchange of information within both the pathological and physiological contexts of cellular behavior, with profound implications for the function of recipient cells, (Yáñez-Mó et al., 2015). Furthermore, it is increasingly apparent that they may contribute to the pathogenesis of many diseases. EVs are divided into different types, namely, exosomes derived from endosomes (exosomes [EXO], 30–150 nm), microvesicles derived from the plasma membrane (microvesicles [MVs], 150–1,000 nm), and apoptotic cells (150–1,000 nm), and apoptotic vesicles (200–5,000 nm) released during apoptosis (Figure 1). These substances with unique biological properties can bind to specific proteins or genes to form complexes that exert therapeutic effects. Owing to the easy recognition of apoptotic vesicles and subsequent clearance by macrophages, this review dealt with EXO and MVs, which depict highly promising functions for clinical translation (Segawa and Nagata, 2015; Théry et al., 2018). EVs have risen as a compelling prospect for cell-free therapeutic schemes to promote tissue regeneration. This is attributed to their remarkable advantages, encompassing superior biosafety, the capacity to traverse biological barriers, and the ability to protect their contents from being degraded (Lazar et al., 2021; Ramasubramanian et al., 2022). Furthermore, owing to their powerful regenerative capacity and easy accessibility, the demand for stem cell-derived EVs is increasing in regenerative medicine (He et al., 2018; Liao et al., 2019; Liu and Su, 2019; Xiao et al., 2019).

However, apart from the established pharmacological role of EVs in intercellular or interorgan communication, recent research has reported that EVs can functionally transport therapeutic biological cargoes (Kamerkar et al., 2017; Poggio et al., 2019; Lathwal et al., 2021). Studies on unmodified EVs *in vivo* and circulation have reported that when administered systemically, EVs are easily recognized by the mononuclear phagocyte system and rapidly absorbed (Wiklander et al., 2015; Parada et al., 2021). Furthermore, the functional modification of EVs using bioengineering techniques is being increasingly recognized as indispensable for their effective adaptation across various clinical therapeutic scenarios (Hao et al., 2020; Cheng and Hill, 2022; Hao et al., 2022). The content of EVs, including those of proteins, lipids, and nucleic acids, can be modified. Many studies have reported that EVs exhibit the feasibility of undergoing functional modification and engineering (Alvarez-Erviti et al., 2011; Liang et al., 2021). EVs have recently emerged as a breakthrough preclinical remedy, and their superior drug delivery capabilities and modifiability have laid a solid foundation for their successful application (Bashyal et al., 2022). Engineered EVs represent a category of modified EVs synthesized by the incorporation of therapeutic molecules using bioengineering techniques. This versatile approach allows for the engineering of EVs both endogenously and exogenously, drawing upon an understanding of their biological structures and biogenesis principles. It capitalizes on the ability of primary EVs to encapsulate therapeutic cargo for precise and targeted delivery (Zipkin, 2019; Zipkin, 2020).

Recently, advances in clinical trials have increased our intuitive comprehension of the translational capabilities of EVs in various therapeutic applications. Among these, EVs derived from MSCs (MSC-EVs) have been observed to be the most prevalent type featured in these trials. Researchers have detected multiple significant EVs, both in terms of potential and clinical significance, as novel targeted delivery systems aimed at facilitating or impeding the introduction of specific bioactive substances into the body to elicit distinct biological effects. According to a survey of clinical trials, MSC-EVs are increasingly being used to treat diseases and deliver therapeutic agents (https://clinicaltrials.gov/ct2/home, accessed 1 May 2023) (Table 1). In three clinical trials (NCT03384433, NCT03608631, and NCT05043181), MSCs were genetically modified to overexpress EVs (Akbari et al., 2022). Furthermore, one clinical trial used plant-derived EVs loaded with curcumin to target normal colon tissues and tumors (NCT01294072). Another trial used autologous stem cell-derived EVs from human deciduous teeth to treat young and immature permanent teeth with pulp necrosis (NCT01814436). At present, MSCs are the most promising parental source of EVs owing to their varied applications, including in tissue engineering and regenerative medicine (Aheget et al., 2020; Dai et al., 2020; Yuan et al., 2021). Table 1 highlights the recent clinical trials involving MSCs or engineered EVs.

**TABLE 1:** Recent observations and clinical trials of EVs derived from mesenchymal stem cells or engineering modification.

Trial identifier (ClinicalTrials.gov)	Trial phase	Condition	Trial purpose or intervention	Start date	Status
NCT04850469	Observation	Severe infection in children	To evaluate the application of MSC-derived exosomes	January 2022	Not yet recruiting (Estimated completion 31 December 2024)
NCT04979767	Observation	Bacterial sepsis	To define immune pathways and identify clinically useful biomarkers	15 April 2021	Recruiting
NCT04270006	Early Phase I	Periodontitis	Treatment with adipose stem cell-derived extracellular vesicles	12 February 2020	Unknown
NCT01294072	Phase I	Colon Cancer	To address the problem of curcumin delivery by using plant exosomes to deliver the drug to colon tumors and normal colon tissue	January 2011	Unknown
NCT03608631	Phase I	Adenocarcinoma	Treatment with mesenchymal stromal cell-derived extracellular vesicles along with KRAS G12D siRNA	27 January 2021	Recruiting (Estimated Completion 30 June 2023)
NCT04664738	Phase I	Skin graft	Treatment with platelet-derived extracellular vesicles	16 March 2021	Unknown
NCT05043181	Phase I	Hypercholesterolemia	To evaluate the safety and preliminary effectiveness of exosome-based older mRNA nano platform for gene therapy in Homozygous individuals	December 2021	Not yet recruiting (Estimated Completion December 2026)
NCT05402748	Phase I/II	Fistula Perianal	Treatment with placenta-MSC-derived exosomes	22 December 2021	Unknown (Estimated Completion 22 March 2023)
NCT05499156	Phase I/II	Perianal Fistula	Treatment with placenta-MSC- derived exosomes	20 January 2022	Unknown
NCT03384433	Phase I/II	Cerebrovascular Disorders	To determine the efficacy of the administration of MSC-derived exosomes in improving disability among patients with acute ischemic stroke	17 April 2019	Unknown
NCT01854866	Phase II	Malignant Pleural Effusion	Treatment with drug-packaging microparticles	May 2013	Unknown
NCT05490173	Not Applicable	Premature Birth	Treatment with exosomes derived from mesenchymal stromal cells	5 October 2022	Not yet recruiting (Estimated Completion 28 December 2026)
NCT01814436	Not Applicable	Dental Pulp Necrosis	Treatment with scaffold-free SHED-derived pellet	February 2013	Unknown

## 2 DSC-EVs in oral and maxillofacial tissue regeneration

### 2.1 Classification of DSC-EVs

**FIGURE 2:**
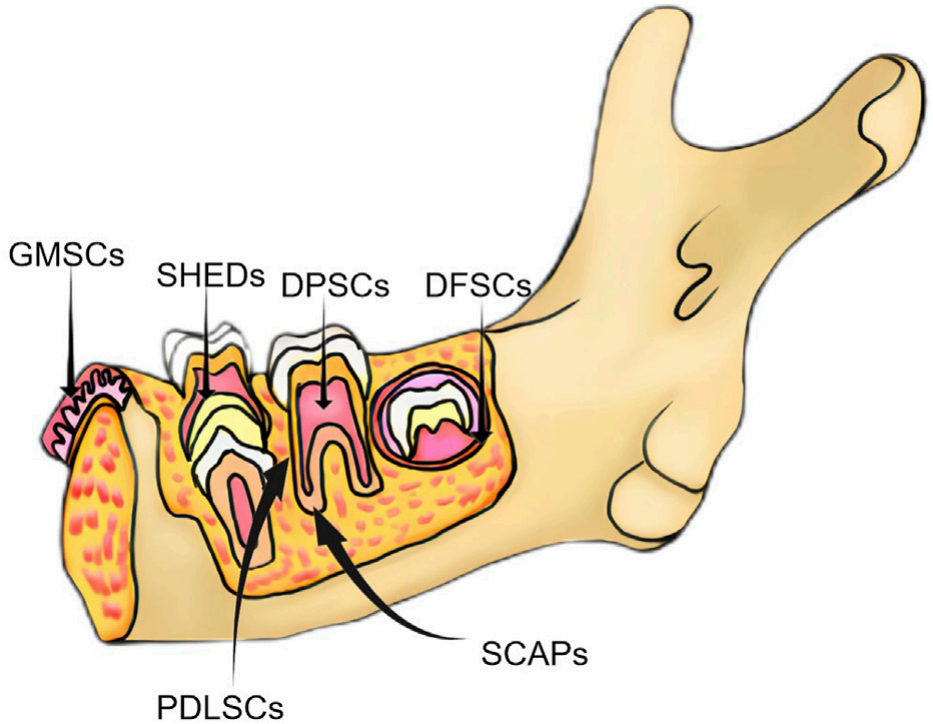
Classification of major dental-derived stem cells.

Dental stem cells can be sourced from various sites within the oral cavity, encompassing the periodontal and inner pulp tissues. Researchers have identified many sites where MSCs are present in the oral cavity, which has led to a genuine interest in the dental tissues and the surrounding soft and complex support structures (Tatullo et al., 2015). Six types of dental tissue-derived stem cells have been isolated and identified in the field of oral tissue regeneration (Figure 2): dental pulp stem cells (DPSCs), stem cells from exfoliated deciduous teeth (SHEDs), stem cells from apical papilla (SCAPs), periodontal ligament stem cells (PDLSCs), dental follicle progenitor cells, and gingival MSCs (GMSCs) (Bakkar et al., 2017). DSCs differ from MSCs derived from the bone marrow (BMMSCs) or adipose tissues. These stem cells hold a distinct advantage due to their embryological origin from the neural crest, which enables them to differentiate into both dental and neural tissues. Notably, their neuro-affinity properties make them particularly intriguing for various applications (Mayo et al., 2014). Human exfoliated deciduous dental stem cells represent an immature subset of MSCs characterized by their remarkable potential for differentiation and potent capacity for proliferation. Additionally, their non-invasive and ethically unproblematic harvest makes them particularly appealing. Nakamura et al. demonstrated that SHEDs are enriched in growth factors, including transforming growth factor β2 (TGF-β2) and fibroblast growth factor-2 (FGF2) (Wu et al., 2019). The ability of apical papilla stem cells, termed SCAPs, to develop into osteoblasts, adipocytes, chondrocytes, and neuronal cells under specific culture conditions underscores their diverse functions. In the context of tooth formation, SCAPs exhibit an increased proliferation rate in comparison with PDLSCs (Chen K. et al., 2013). They have the capacity to develop into odontogenic and osteogenic progenitor cells, adipocytes, and neuronal cells (Yang B. et al., 2017). On the other hand, PDLSCs can undergo differentiation into collagen-forming cells, adipocytes, and odontogenic osteoblasts *in vitro*. They also exhibited the capacity for regeneration of odontoblasts and periodontium in various animal models (Seo et al., 2004). The dental follicle, which envelops the developing tooth as a connective capsule, houses a subset of MSCs called DFPCs. DFPCs possess a remarkable versatility in differentiation, with the capacity to transform into osteoblasts, adipocytes, and neural cells. Notably, DFPCs are capable of initiating neural differentiation and contributing to the regeneration of periodontal structures and bone tissue (Morsczeck et al., 2005). GMSCs are readily harvested from gingival tissues of the oral cavity with minimal damage to the organism. Furthermore, GMSCs can form connective tissue-like structures *in vivo*, and *in vitro*, they can form mineralized nodules, adipose, and chondrocyte-like matrices when cultured with different growth factors (Ge et al., 2012). These cells are superior compared to the widely acclaimed BMSCs in terms of regenerative potential (Tomar et al., 2010). DSC-EVs are involved in angiogenesis, anti-inflammation, intercellular communication, immunomodulation, osteogenesis, neuron nourishment, and promoting apoptosis in tumor cells. Owing to their accessibility, modifiability, and high proliferative capacity, DSCs have been extensively studied for regulating the development of dental, skeletal, and salivary gland tissues, repairing trauma to maxillofacial tissues, and regenerating pulpal and periodontal tissues (Li et al., 2018; Mai et al., 2021) (Table 2).

**TABLE 2:** Isolation, characterization, and functions of DSC-derived EVs.

Potential application types	Origin	EV isolation methods	Identification methods	Administration	Recipients	Ref
Bone and vascular regeneration	SHED	The CM was centrifuged at 300 ×g for 10 min, 2,000 ×g for 15 min, and 10,000 ×g for 30 min, and concentrated using ultrafiltration at 100,000 ×g for 1 h	■ TEM	In vitro/in vivo	HUVEC, BMSC/SD Periodontal Defect Rat Model	Wu et al. (2019)
			■ NTA			
			■ BCA			
			■ WB(CD81, CD9 and TSG101)			
		The CM was centrifuged at 300× g for 10 min, 2,000 ×g for 10 min, 20,000 ×g for 30 min, and 100,000 ×g for 70 min	■ TEM	In vitro	PDLSCs	Wang et al. (2020)
			■ BCA			
			■ NTA			
			■ WB (CD9,CD63, TSG101 and Calnexin)			
	GMSC	ExoQuick-TC exosome isolation reagent	■ DLS	In vitro/in vivo	hGMSCs/Cranial injury rat model	Diomede et al. (2018b)
			■ AFM			
			■ WB (CD9, CD63,CD81, and TSG101)			
	SCAP	The CM was ultracentrifuge at 3,000 ×g for 20 min, 20,000 ×g for 30 min, and 120,000 ×g for 2 h	■ TEM	In vitro/in vivo	HUVEC/palatal gingival defect mouse model	Liu et al. (2021)
			■ NTA			
			■ WB (CD9,CD63, and Alix)			
	PDLSC	The CM was centrifuged at 3,000 ×g for 20 min; 16,500 ×g for 20 min; filtered using a 0.2-µm filter; and ultracentrifuged at 100,000 ×g for 70 min	■ NTA	In vitro	hPDLSCs	Xie et al. (2021)
			■ Flow cytometry			
			■ TEM			
			■ WB(CD63 and CD81)			
		Centrifugation for 10 min at 500 ×g, 30 min at 16,000 ×g, followed by ultracentrifugation for 70 min at 150,000 ×g	■ Flow cytometry	In vitro	HUVECs	Zhang et al. (2020b)
			■ TEM			
			■ WB (TSG101 and CD63)			
		ExoQuick-TC exosome isolation reagent	■ DLS	In vitro/in vivo	hPDLSCs/rat calvarial defect model	Pizzicannella et al. (2019)
			■ AFM			
		ExoQuick-TC exosome isolation reagent	■ DLS	In vitro/in vivo	hPDLSCs/rat calvarial defect model	Diomede et al. (2018a)
			■ AFM			
		Centrifugation at 300 ×g for 10 min, 2000 ×g for 10 min; 20,000 ×g for 30 min, followed by ultracentrifugation at 100,000 ×g for 70 min	■ TEM	In vitro	hPDLSCs	Čebatariūnienė et al. (2019)
			■ NTA			
			■ WB(CD63)			
	DPSC	The CM was centrifuged at 300 ×g for 10 min, 2,000 ×g for 10 min, and 10,000 ×g for 30 min, followed by ultracentrifugation twice at 100,000 ×g for 70 min	■ TEM	In vitro/in vivo	HUVEC/full-thickness skin defect mouse model	Zhou et al. (2020)
			■ NTA			
			■ WB (Alix,HSP70, CD9, CD63 and CD81)			
			■ BCA			
		The CM was centrifugated at 500 ×g for 10 min at 4°C; 2,000 ×g for 10 min; 10,000× g for 1 h at 4°C; filtered using a 0.22-μm filter; and ultracentrifuged at 100,000 ×g for 2 h	■ TEM	In vitro	Schwann cells	Li et al. (2021)
			■ NTA			
			■ WB			
		Exosome isolation reagent	■ TEM	In vitro	hBMMSCs	Ivica et al. (2020)
			■ WB (CD9, and Grp94)			
Periodontal regeneration	DPSC	The CM was centrifuged at 300 ×g for 10 min and 16, 500 ×g for 20 min, followed by ultracentrifugation at 120,000 ×g for 2.5 h at 4°C	■ TEM	In vitro/in vivo	Macrophages/periodontitis mouse model	Shen et al. (2020)
			■ NTA			
			■ WB (CD9, HSP70 and TSG 101)			
			■ Flow Cytometry (CD 63 and CD 81)			
		Exo-spin exosome isolation reagent	■ WB (CD9 and CD63)	In vitro	hDPSCs	Hu et al. (2019)
			■ TEM			
		ExoQuick-TC exosome isolation reagent	■ WB (CD63 and CD9)	In vitro	DPSC/hMSCs	Huang et al. (2016)
			■ TEM	/in vivo		
	GMSC	MagCapture TM exosome isolation kit	■ TEM	In vitro/in vivo	Macrophage/ligature-induced periodontitis mouse model	Nakao et al. (2021)
			■ NTA			
			■ WB (CD9, CD63 and CD81)			
		The CM was centrifuged at 300 ×g for 10 min and 10,000 ×g for 40 min; filtered through a 0.22-mm filter; and ultracentrifuged for 70 min at 100,000 ×g twice	■ NTA	In vitro	GMSCs and PDLSCs	Hu et al. (2023)
			■ TEM			
			■ WB(CD9, CD63, ALIX, and HSP70)			
	DFPC	Exosome isolation reagent	■ TEM	In vitro/in vivo	PDLCS/Periodontitis rat model	Shi et al. (2020)
			■ NTA			
			■ WB (CD63 and TSG101)			
	SHED	The CM was centrifuged at 300 ×g for 10 min, 2,000 ×g for 10 min, 20,000 ×g for 30 min, and 100,000 ×g for 70 min	■ TEM	In vitro	PDLSCs	Wang et al. (2020)
			■ BCA			
			■ NTA			
			■ WB (CD9,CD63, TSG101 and Calnexin)			
		The CM was centrifuged at 300 ×g for 10 min, 2000 ×g for 10 min, and 10,000 ×g for 60 min filtered through a 0.22-µm filter; and ultracentrifuged at 100,000 ×g for 70 min twice	■ TEM	In vitro/in vivo	BMSCs/OVX mouse model	Wei et al. (2020)
			■ WB (CD63)			
Pulp regeneration	DPSC	Exo-spin (Cell Guidance) exosome isolation reagent	■ WB (CD9 and CD63)	In vitro	hDPSCs	Hu et al. (2019)
			■ TEM			
		ExoQuick-TC exosome isolation reagent	■ WB (CD63 and CD9)	In vitro/in vivo	DPSC/hMSCs	Huang et al. (2016)
			■ TEM			
	SHED	The CM was centrifuged at 2,000 ×g for 10 min and 10,000 ×g for 30 min, followed by ultracentrifugation at 100,000 ×g for 70 min twice	■ TEM	In vitro/in vivo	HUVECs/SHEDs	Wu et al. (2021a)
			■ NTA			
			■ WB(CD9, CD63, CD81)			
Dentinogenesis	SCAP	The CM was centrifuged at 3,000 ×g for 20 min, 20,000 ×g for 30 min, and 120,000 ×g for 2 h	■ TEM	In vitro/in vivo	BMMSCs/dentinogenic cell (in nude mice)	Zhuang et al. (2020)
			■ NTA			
			■ BCA			
			■ WB (CD9, and Alix)			
	DPSC	The CM was centrifuged at 500 ×g for 10 min, 2,000 ×g for 10 min, 10,000 ×g for 1 h; filtered through 0.22-µm filter; and ultracentrifuged twice at 100,000 ×g for 2 h and 70 min at 4°C	■ BCA	In vitro	SCs	Li et al. (2021)
			■ WB (alix, CD9, CD63 and GM130)			
			■ TEM			
			■ NTA			
		The CM was centrifuged at 300 ×g for 10 min and 2,000 ×g for 10 min; filtered through a 0.22-µm filter; centrifuged at 4,000 ×g for 10 min; ultracentrifuged at 4,000 ×g to 200 μL and 100,000 ×g for 70 min twice	■ NTA	In vitro/in vivo	DPSC/Rat Pulpotomy Model	Swanson et al. (2020)
			■ BCA			
			■ WB(CD9, CD63, and CD81)			
Nerve tissue regeneration	GMSC	ExoQuick-TC exosome isolation reagent	■ BCA assay	In vitro/in vivo	SCs/Nerve damage mouse model	Mao et al. (2019)
			■ NTA			
			■ WB (CD 63 and CD9)			
	SHED	The CM was centrifuged at 300 ×g for 10 min, 2000 ×g for 10 min, and 20,000 ×g for 30 min, followed by ultracentrifugation at 100,000 ×g for 70 min twice	■ WB	In vitro	Human neural stem cells	Jarmalavičiūtė et al. (2015)
		The CM was centrifuged at 300 ×g for 10 min, 2,000 ×g for 10 min, and 20,000 ×g for 30 min, followed by ultracentrifugation at 100,000 ×g for 70 min twice	■ NTA	In vivo	PD rat model	Narbute et al. (2019)
			■ TEM			
		ExoQuick Reagent Kits	■ WB(CD81, CD63, and CD9)	In vivo	TBI rat model	Li et al. (2017)

Abbreviations: CM, conditional medium; TEM, transmission electron microscope; NTA, nanoparticle tracking analysis; DLS, dynamic light scattering; AFM, atomic force microscope.

### 2.2 Bone and vascular tissue regeneration

Bone tissue regeneration involves the coordinated regulation of various cell types, encompassing osteoblasts, osteoclasts, chondrocytes, and endothelial cells. The crucial stages of bone regeneration are angiogenesis and osteogenesis (Qin et al., 2016). Angiogenesis is essential for bone healing because, in this process, new blood vessels are formed that provide nutrients and oxygen to the surrounding cells. A study showed that a conditioned medium deprived of EVs inhibited angiogenesis (Shabbir et al., 2015). Wu et al. reported that SHED-EVs promoted the angiogenesis as well as the proliferative and migratory capacities of human umbilical vein endothelial cells (HUVECs). This effect was mediated via the 5′AMP-activated protein kinase pathway. Additionally, they promoted the neovascularization and the formation of new bone in a rat model of periodontal bone defects (Wu et al., 2019). Diomedee et al. inoculated the gingival tissues of volunteers with 3D polylactic acid scaffolds, and human gingival MSC (hGMSC)-derived EVs were found to increase calcium deposition as well as elevate RUNX2 and BMP2/4 gene expression levels and protein levels. Furthermore, EVs in PLA scaffolds significantly promoted bone and angiogenesis in rats with cortical cranial defects (Diomede et al., 2018b). Moreover, these EVs promoted the regeneration of palatal gingival tissues. Another study reported that SCAP-EVs remarkably enhanced the migratory and angiogenic capacities of HUVECs (Liu et al., 2021). Xian et al. reported that DPSC-EVs enhanced vascular endothelial cells’ proliferation and angiogenic capacities and verified that these effects were achieved via the p38 mitogen-activated protein kinase (MAPK) signaling pathway (Xian et al., 2018). In another study, when Schwann cells, the primary source of odontoblasts, were co-cultured with EVs from lipopolysaccharide (LPS)-pretreated hDPSCs, they exhibited enhanced migration and osteogenic differentiation abilities (Li et al., 2021). Ivica et al. observed that hDPSC-derived small EVs encapsulated in fibrin gels promoted the migration and proliferation of human BMMSCs (hBMMSCs) (Ivica et al., 2020). Moreover, Huang et al. revealed that miR-223-3p may be pivotal in the mechanism by which LPS–hDPSCs–EXo promotes angiogenesis in HUVECs (Huang et al., 2021). Furthermore, Zhou and Pizzicannel revealed that hDPSC-EVs were critically involved in promoting angiogenesis and wound healing in the constructed mouse skin defect and rat cranial defect models, respectively. These EVs were also observed to enhance vascular and bone formation (Pizzicannella et al., 2019; Zhou et al., 2020).

### 2.3 Periodontal tissue regeneration

Periodontal tissues comprise the gingiva, periodontal ligament, dental bone, and alveolar bone. Periodontal tissue health is crucial to the overall masticatory function. Periodontal diseases leading to resorption or loss of the alveolar ridge can result in symptoms such as pain and bleeding. The alveolar ridge plays a vital function in providing both physical and mechanical support to the teeth. Bone loss and neovascularization are the main pathological changes during periodontal disease development. Multiple factors, including bone metabolism, osteoblasts, and matrix metalloproteinases, regulate periodontal tissue repair and reconstruction during periodontal disease development. The periodontal treatment promotes periodontal tissue regeneration by functionally reattaching the periodontal ligament to the new dental and alveolar bone (Liu et al., 2019). Traditional periodontitis therapies include scaling, periodontal scraping, systemic and topical antibiotics, and oral disinfectants. Although these treatment strategies can inhibit the progression of periodontitis in the short term, long-term efficacy regarding the condition of the patient and initiatives should be evaluated. Therefore, developing new strategies to effectively treat periodontitis and achieve periodontal tissue regeneration is imperative (Elashiry et al., 2021). Hu et al. reported that gingiva-derived MSC (GMSC)-EVs exerted an immunomodulatory effect by promoting the expression of osteogenic differentiation-related factors within PDLSCs, irrespective of whether they were in normal and inflammatory environments. Furthermore, several other studies have corroborated that GMSC-EVs possess the ability to inhibit Porphyromonas gingivalis LPS-induced nuclear factor kappa B (NF-κB) pathway activation. This inhibition triggers the activation of the Wnt/β-catenin pathway, indicating the presence of a mutually exclusive regulatory mechanism between the NF-κB and Wnt/β-catenin pathways in the peripheral inflammatory environment. GMSC-EVs were found to excel in cross-regulating these two pathways, underscoring their remarkable regulatory ability (Hu et al., 2023). Likewise, Nakaod et al. observed that locally injected GMSC-EVs considerably decreased periodontal bone resorption in a ligation-induced mouse periodontitis model (Nakao et al., 2021). Moreover, Shen et al. documented that DPSC-EVs promoted periodontal epithelial healing and alveolar bone reconstruction in a periodontitis mouse model, primarily through the delivery of miR-1246 (Shen et al., 2020). Shi et al. conducted research involving the extraction of LPS-pretreated dental follicle cell-derived EVs. The resulting data implied that these EVs promoted the osteogenic differentiation potential as well as the proliferative and migratory capacities of periodontal ligament-derived stem cells (hPDLSCs) from patients with periodontitis *in vitro*. *In vivo*, they enhanced directed periodontal ligament formation and promoted the formation of periodontal bone. These EVs were observed to be linked to decreased TRAP-positive osteoclast and RANKL/OPG expression (Shi et al., 2020). Wang et al. we’re pioneers in documenting the role of SHED-EVs in promoting the osteogenic differentiation of PDLSCs *in vitro* by inducing the Wnt/β-catenin and BMP/Smad pathways. This discovery carries notable clinical implications (Wang et al., 2020). In a separate study, Wei et al. achieved the successful rescue of bone loss in mice with periodontitis by locally injecting SHED-EVs. Their *in vitro* experiments revealed that SHED-EVs exhibited a multi-faceted effect. They not only stimulated the osteogenesis and proliferation of cells but also effectively attenuated adipogenesis and inflammatory cytokine secretion in mouse bone marrow stromal stem cells (mBMSCs) (Wei et al., 2020). The acquired data provide insights for developing novel therapeutic strategies for periodontitis and promoting periodontal tissue regeneration.

### 2.4 Pulp regeneration

As a highly vascularized and innervated tissue, the pulp has various functions, such as triggering immune responses to microbial insults and injuries, providing neuronal sensitivity, and promoting repair and regeneration in response to mechanical stimuli. The loss of the pulp tissue leads to the loss of tooth vitality. The root canal remains the preferred method for treating permanent necrotic teeth (Cvek, 1992). Nevertheless, new pulp regeneration therapies should be explored to maintain the biological function of the tooth. An EV-mediated pulp tissue regeneration method could serve as an effective alternative to the existing root canal treatment. Hu et al. reported that DPSC-EVs promoted the odontogenic differentiation of hDPSCs via the tumor growth factor (TGF)β1/SMAD pathway (Hu et al., 2019). Meanwhile, using a tooth fragmentation mouse model, Wu et al. demonstrated the regeneration and angiogenic ability of DSC-EVs *in vivo*. They reported that they further promoted SHED differentiation toward endothelium and increased HUVEC angiogenesis *in vitro*. Furthermore, they reported that miR-26a-rich SHED-EVs promoted angiogenesis via the TGF-β and SMAD2/3 signaling pathways, enhancing pulp tissue regeneration (Wu M. et al., 2021). Huang et al. reported that DPSCs and BMSCs endocytosed DPSC-EVs in a dose-dependent manner and activated the p38 MAPK pathway, thereby inducing DPSC differentiation and tissue regeneration. Furthermore, they validated the ability of DPSC-EVs to stimulate pulp-like tissue regeneration while considerably increasing the dentin matrix acidic phosphoprotein 1 (DMP1) and dentin sialophosphoprotein (DSPP) levels at the soft tissue–dentin junction in an *in vivo* root section model (Huang et al., 2016). The resulting data of this research provide a solid theoretical foundation for the potential application of EV therapy in promoting pulp regeneration.

### 2.5 Dentin regeneration

Progression infectious disease that causes hard tissue loss in the teeth, leading to pulp inflammation, thereby affecting tooth regeneration. During dental caries development, dentin-forming cells produce reactive dentin to inhibit disease progression (Farges et al., 2015). SCs primarily differentiate into dentin-forming cells after migrating to the damaged sites during tooth development and regeneration (Kaukua et al., 2014). Furthermore, Li et al. reported that hDPSC-derived EVs stimulated dentin salivary protein production and SC mineralization while increasing the expressions of DSPP, DMP1, osteocalcin, and RUNX2 genes in an isolated environment, thereby mediating the odontogenic differentiation of SCs (Li et al., 2021). Additionally, Zhuang et al. revealed that SCAP-EVs could promote the differentiation ability of dentin tissues and adult dentin cells. Moreover, SCAP-EVs exerted an effect on BMMSC proliferation *in vitro* but considerably modulated their dentin formation ability by increasing DSPP levels in BMMSC and forming mineralized nodules (Zhuang et al., 2020). Swanson et al. reported that DPSC-EVs can promote hDPSC migration and induce the mineralization process in a dose-dependent manner. Based on these findings, they developed a controlled-release platform that can be implanted into the defective sites without exogenous SC transplantation, thereby stimulating the endogenous DPSC migration from the pulp chamber and directing their differentiation fate toward a certain regenerative outcome. Using this EV delivery platform, EVs derived from hDPSCs and odontoblasts were released at the pulp interface on exposure to the body. They induced the migration and differentiation of DPSCs into dentin cells, producing reactive tertiary dentin bridges that may effectively prevent tooth necrosis (Swanson et al., 2020).

### 2.6 Nerve tissue regeneration

Annually, a significant number of cases involving neurological disorders, encompassing both central and peripheral nervous system disorders, are documented worldwide. Nerve damage resulting from various factors such as trauma and tumors is also prevalent, posing a substantial challenge due to the lack of effective treatment schemes. The impact of these conditions extends beyond patients themselves, affecting their families and placing a heavy burden on society (Taylor et al., 2008; Asplund et al., 2009). While autologous nerve grafting remains the established standard for rectifying nerve defects, the quest for innovative alternatives is imperative. SC-based therapies, particularly those using DSCs, offer new hope for treating neurological disorders. Reportedly, DPSCs are more suitable for treating neurodegenerative diseases than BMMSCs or adipose tissue-derived MSCs of the mesoderm (Wang et al., 2019). DSCs arising from the neural crest share features similar to those derived from the neural cells. They exhibit the capability to differentiate into various functional units, including neurons, astrocytes, and fibroblasts, which hold promise as ideal sources for cellular nerve regeneration and repair (Li et al., 2018). Among all DSCs, SCAPs exert the strongest neurotrophic effects, suggesting they may be the best EV progenitor cell source for peripheral nerve repair (Kolar et al., 2017). Mao et al. simulated clinical nerve repair *in vivo* and found that hGMSC-EVs actively facilitated axonal repair, functional recovery, and nerve regeneration in extrusion-injured mice. Furthermore, the propagation and migration of SCs were stimulated in an *ex vivo* setting (Mao et al., 2019). Li et al., using a traumatic brain injury rat model, concluded that SHED-EVs were involved in the recovery of motor function and reduced cortical damage in rats by promoting the polarized movement of microglia (Li et al., 2017). In the AKVILE study, SHED-EVs could inhibit approximately 80% of 6-hydroxydopamine (OHDA)-induced apoptosis in dopaminergic neuronal cells, suggesting the potential neuroprotective effect of SHED-EVs on human dopaminergic neurons (Jarmalavičiūtė et al., 2015). Narbute et al. achieved a significant milestone by demonstrating the therapeutic efficacy of SHED-EVs in Parkinson’s disease rat models with 6-OHDA-induced medial forebrain bundle injury for the first time (Narbute et al., 2019). A. Nasirishargh et al. found that multiple exosomal miRNAs have been identified to have neuroprotective effects by promoting neurogenesis, neurite remodeling, and survival (Lazar et al., 2022). These studies contribute to a solid theoretical foundation for EV-based treatment of neurological diseases and neural tissue regeneration (Nasirishargh et al., 2021).

Overall, altering the culture conditions of donor cells, such as hypoxic or inflammatory conditions or the differentiation of donor cells, may enhance the beneficial therapeutic effects of secreted EV. However, pretreated mother cells have restricted control over the specific cargoes of these enhanced EVs as only naturally occurring cargo molecules can be enriched through these methods.

## 3 EV engineering strategies

### 3.1 Feasibility

**FIGURE 3:**
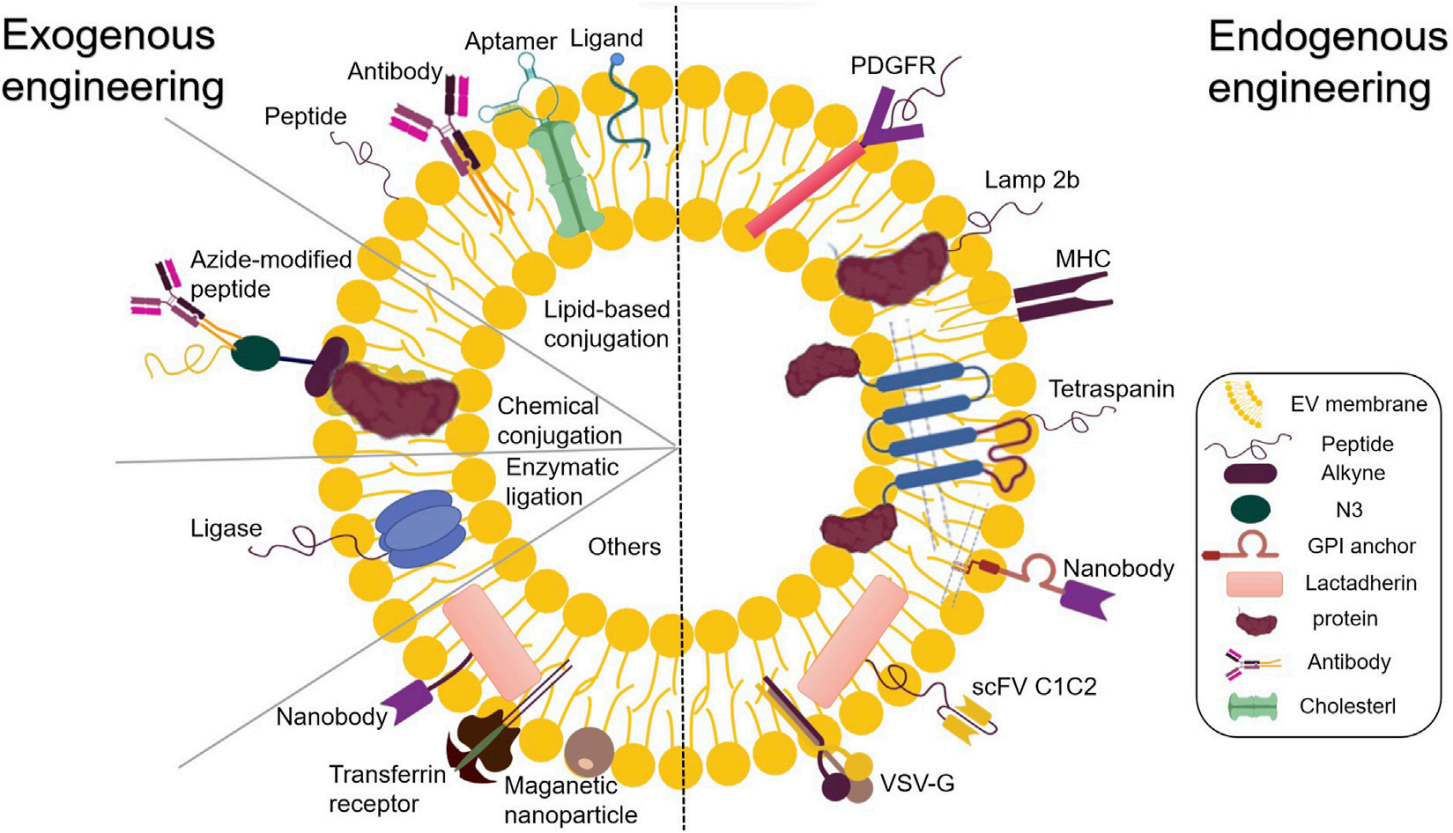
Endogenous and exogenous EV engineering strategies for therapeutic cargo loading.

Alongside the established pharmacological role of EVs in intercellular or interorgan communication, many recent studies have reported their functional ability to transport therapeutic cargo. EVs can be designed to transport desired components, including proteins, antibodies, drugs, and RNA of interest. Furthermore, studies have reported successfully incorporating diverse small molecules and clinical drugs into EVs (Kamerkar et al., 2017; Poggio et al., 2019; Lathwal et al., 2021). EVs exhibit therapeutic potential for various diseases, opening endless possibilities for the medical community. After their isolation, EV components can be used for endogenous and exogenous engineering at the cellular level (Hood, 2016). The present review deals with the approach to functional cargo modification (both endogenous and exogenous) of EVs. Engineering techniques for endogenous modification mainly involve the genetic engineering of source cells for overproducing proteins of interest and their fusion with EV biogenesis-related proteins, thereby exploiting the inherent ability of EVs to incorporate functional cargoes. Over the past decade, numerous endogenous engineered stents have been tested for EV cavity and surface engineering (Wiklander et al., 2019) (Figure 2). On the other hand, exogenous modification is achieved by incorporating functional cargoes into EVs by using cell membrane surface receptor proteins, ligands, and other molecular properties to achieve specific targeting (Rädler et al., 2023) (Figure 3).

### 3.2 Loading of endogenous functionalized cargo

#### 3.2.1 Genetic and EV surface protein engineering

The utilization of miRNAs holds tremendous potential in the field of regenerative medicine. Considering the selective packaging of miRNAs within EVs by secretory cells, there has been extensive investigation into MSCs enriched for certain miRNAs *via* transfection or transduction processes. Genetic engineering strategies offer a means to generate functional EVs from cells expressing biomolecules either in the lumen or on the surface of the EV. RNA and protein sequences can be efficiently introduced into these EVs through transfection using synthetic oligonucleotides or by being expressed from a plasmid backbone (Ohno et al., 2013; Kooijmans S. A. A. et al., 2016). Parental cells can be transfected with therapeutic molecules, including RNA, proteins, ligands, and receptors, for overexpression. These molecules subsequently reach the lumen or onto the surface of EVs, presenting their unique biological properties (Lin et al., 2018; Mendt et al., 2018). This facilitates the entry of exogenous genes into the host cell. Advancements in the elucidation of the EV biogenesis pathways have improved the understanding of precise RNA sorting mechanisms, dramatically accelerating the development of EV bioengineering strategies. An integral aspect of this endeavor is the utilization of EVs as nanocarriers to construct corresponding transport systems. One possible approach for regulating the loading of nucleic acid cargoes into EVs is to fuse nucleic acid-binding proteins with EV-sorting proteins. The efficient loading of nucleic acids into EVs during biogenesis is achieved by the co-expression of the fusion protein with nucleic acids displaying compatible binding motifs (Liang et al., 2022). This approach has been successfully applied to short and long RNA sequences (Kojima et al., 2018; Wang et al., 2018). Targeted therapeutic mRNA expression within EV-derived cells allows the introduction of therapeutic proteins in EVs (Zickler and El Andaloussi, 2020).

Apart from gene transfer, another mechanism for delivering the regenerative potential of EVs involves the delivery of proteins with the capacity to influence target cells. EV can deliver proteins capable of regulating multiple aggregation pathways in its lumen (Qiu et al., 2019). V components can be engineered endogenously within the cell or exogenously after isolation (Mentkowski et al., 2018). The former can be modified by chemical engineering strategies combining metabolic engineering and click chemistry (You et al., 2021). It is worth highlighting that these covalent chemical reactions render EV functionalization more stable in comparison to non-covalent engineering methods (Armstrong et al., 2017).

The EV surface is rich in glycosylphosphatidylinositol (GPI)-anchored proteins or various transmembrane proteins, which can serve as drug carriers to augment the impact of the drug on the regulation of specific genes expressed on the target cell membrane. Different types of molecules can be used to modify these proteins. The various effector functions of these proteins, including but not limited to target cell recognition (Hoshino et al., 2015), ligand signaling (Chen et al., 2018), and induction of toxins, biological agents, and viral receptors (de Carvalho et al., 2014; Keller et al., 2020), result in a spectrum of bodily functions. Considering the presence of transmembrane molecular complexes in various forms within tissues, it is imperative to adopt a tailored approach for interactions among these complexes to achieve specific objectives. This review substantiated the feasibility of various surface-engineered scaffolds by systematically comparing EV-related transmembrane proteins. Tetraspanins (cluster of differentiation [CD]63, CD9, and CD81), lysosome-associated membrane protein (LAMP)-2b, GPI, platelet-derived growth factor receptors, and cadherins (the C1C2 structural domain) are essential in mediating cytoskeletal integration for maintaining tissue homeostasis. Prostaglandin F2 receptor negative regulator is widely used for transmembrane modifications. These transmembrane proteins play crucial roles in different tissues, mediating drug transport, promoting angiogenesis, and inducing inflammatory responses. The complex interactions among transmembrane proteins render their precise regulation difficult. Less research has been done to modify the therapeutic protein content of EV through donor cell modification. To modify the cargo of therapeutic proteins within EVs, a similar strategy to that used for miRNAs can be applied (Nagelkerke et al., 2021). Utilizing genetic engineering techniques, it is possible to create a modified exosomal membrane protein with specific signaling or homing properties. This is achieved by expressing a fusion cassette in parental cells through a gene transfer vector, such as a retroviral or lentiviral vector. Consequently, transduced cells produce EVs and express the desired polypeptide on their surface. It has been shown that cells expressing the glycoprotein (G protein) of vesicular stomatitis virus (VSV) can efficiently release VSV-G into cell-derived EVs (Montagna et al., 2018). These VSV-G-modified EVs can be loaded with protein cargo, which enhances their delivery capacity (Meyer et al., 2017). Recombinant proteins with different activities can be identified by evaluating the effective integration of the exogenous target protein or antibody into the membrane of the recipient cell. All four transmembrane proteins of EVs with the surface domains can be used for fusion with the targeting/therapeutic molecules (Corso et al., 2019; Gupta et al., 2021).

However, endogenous engineering strategies are unsuitable for personalized medicine applications because of the unfeasibility of applying this approach to the cells of the patient. Moreover, the approach is laborious and time-consuming, making it challenging to produce on a large scale, and only genetically encodable functional moieties can be used. In addition, due to the wide variety of proteins involved in EV biogenesis and cargo loading, it is challenging to determine a generic strategy for loading biotherapeutic cargo into EVs as well as the exact expression of the functional fraction in the final isolated EVs and the number of functional EVs.

#### 3.2.2 Others

Parental cell co-incubation: This method involves co-culturing the drug with parental cells, allowing the drug to cross the cell membrane. The drug-laden EVs can be isolated after they are secreted by cells. However, the loading efficiency is typically low and strongly dependent on the gradient of drug dose, drug properties, and parental cell types (Smyth et al., 2015; Kanchanapally et al., 2019). To overcome this challenge, it has been evidenced that low currents and UV irradiation of cells may increase the rate at which nanoparticles or drugs are loaded into EVs (Ye et al., 2018; Fukuta et al., 2020).

### 3.3 Loading of exogenous functionalized cargo

#### 3.3.1 Parental cell–EV co-incubation method

Isolated EVs are co-incubated with high concentrations of drugs, following which the drug passes through the EV membrane in a concentration-dependent manner. This is a simple method that helps in maintaining membrane integrity. This passive loading method is advantageous for loading hydrophobic compounds (Kugeratski et al., 2021; Tang et al., 2021). Kim et al. co-incubated paclitaxel with another anticancer drug, indocyanine green (ICG), to obtain dual-loaded EVs (Kim et al., 2022). However, this strategy is limited due to the dependence of its efficacy on the lipophilicity and concentration gradient of the drug, making it less efficient for drug loading; for example, paclitaxel and curcumin have different loading efficacies when co-incubated under the same conditions (Sun et al., 2010; Wei et al., 2019).

#### 3.3.2 Electroporation

Electroporation is primarily applied to nucleic acids (Donoso-Quezada et al., 2020; Elsharkasy et al., 2020). An electric field can be applied to EVs suspended in a conductive solution to create pores in the EV membranes, thereby facilitating the loading of siRNAs or miRNAs, followed by restoring vesicle membrane integrity and forming drug-laden vesicles (Alvarez-Erviti et al., 2011). Kasper observed that electroporation did not alter the ability of adipose tissue-derived EVs to endogenously stimulate glioblastoma cell proliferation. However, it did lead to unfavorable morphological alterations, including EV aggregation. To counter this problem and maintain EV structural integrity, they successfully optimized a buffer system containing alginate and achieved the desired results (Johnsen et al., 2016).

#### 3.3.3 Sonication

This method uses the principle of ultrasound to induce deformations in the EV membrane, allowing for the entry of drugs inside the EVs. This method has been used in the treatment of cancer and diabetes. The underlying principle is that the drug can flow into the EVs because of the ultrasound-induced deformations in the EV membrane. In particular, the EVs are mixed with the drug and treated in a sonic disintegrator (Kim et al., 2016). Haney successfully used this method to infuse peroxidase into EVs, increasing peroxidase activity in the cells (Haney et al., 2015).

#### 3.3.4 Extrusion and hypotonic dialysis

This method can be utilized for the preparation of biomaterials or drug carriers, including tissue engineering scaffolds and immunomodulators. The process of extrusion entails mixing EVs with a drug, introducing this mixture into a syringe-based lipid compressor, and extruding it from a membrane with a 100–400 nm pore size. Hypotonic dialysis is achieved by transferring EVs and drugs into the dialysis membranes, placing them in a phosphate buffer, and stirring the mixture at room temperature. The EV membrane is disrupted during extrusion/dialysis and mixed violently with the drug, allowing the loading of the drug into the EV (Fuhrmann et al., 2015).

#### 3.3.5 Freeze-thaw cycles

In this method, the drug is mixed with EVs and frozen quickly, followed by thawing at room temperature. The freeze-thaw cycle is repeated at least three times to ensure drug encapsulation. The hydrophobic nature of lipid molecules prevents the direct loading of lipids into EVs. Yuko suggested a novel method to engineer hybrid EVs by fusing EV membranes with liposomes (Sato et al., 2016).

#### 3.3.6 Saponins

Saponins are natural glycosides with various pharmacological properties. They are known to exert adverse effects on cells; however, their protective effects, such as tumor growth inhibition and anticoagulation, have also been reported. Saponins induce the formation of tiny pores in lipid membranes, and thus, they can be used as membrane permeabilizers. This process, known as saponification, allows hydrophilic nonpermeable drugs or other molecules to enter EVs (Podolak et al., 2010; Hettich et al., 2022). In contrast to ultrasound, compression, and HP, this method preserves the standard structures of the vesicles without causing changes in size distribution or zeta potential of the loaded EVs. Furthermore, this technique enables the loading of lipid molecules into EVs without special devices. However, saponins’ *in vivo* hemolytic properties must be fully considered when delivered as drugs to ensure safety and efficacy (Haney et al., 2021).

#### 3.3.7 Exogenous modification of EV surfaces

In addition to the EV transporting proteins in its lumen capable of regulating multiple aggregation pathways, another mechanism *via* which EVs influence target cells is by means of proteins present on their surface. These surface proteins enable EVs to dock with receptors located on the surface of the target cell (French et al., 2017). In addition to chemical engineering strategies targeting the source cell membrane before EV isolation, EV membrane modification directly after EV secretion is another critical exogenous approach to EV functionalization by covalent or noncovalent methods (Liang et al., 2021). Typically, the former approach applies bioconjugation, amidation, aldehyde-amine condensation, and click chemistry to link molecules on the EV surface through chemical bonding, while the latter approach modifies EV membranes through hydrophobic insertion, receptor-ligand binding, fusion, and multivalent electrostatic interactions (Yang Y. et al., 2017; Tian et al., 2018; Wu P. et al., 2021; Guo et al., 2021). Exogenous modifications to EV surfaces are used to optimize and improve their surface structures and are performed using chemical methods. Polymers exhibiting specific biological activities are extracellular constructed, and exogenous molecules are attached to the cell membranes via chemical bonding or self-assembly. EVs are subjected to novel functionalization with artificially designed materials that have been synthesized to enhance their performance. These materials exhibit different properties enabling various biological functions, including cell recognition and drug delivery. The physical coupling of targeting ligands to surface proteins is a crucial step in this method. The integration of these molecules into EV membranes via binding affinity antibodies leads to the formation of complexes exhibiting specific biological activities. Highly selective compounds with specific affinities can be obtained by attaching a peptide or antigen fragment with a specific recognition site to the receptor molecule (Hao et al., 2022). Targeted ligands can be attached to isolated EVs in a controlled manner, which is impossible with parental cell modification methods. Methods for targeting peptides and antibodies are commonly used to achieve effective targeting after EV isolation. Isolated EVs are exogenously functionally modified using chemical reactions (click chemistry and noncovalent reactions) to attach therapeutic molecules to their surfaces. These methods involve either the chemical modification of alkynes that are EV membrane proteins or the incorporation of alkyne-modified lipids into EV membranes (Smyth et al., 2014; Tian et al., 2018; Zhu et al., 2019).

Using copper-free click chemistry, EVs containing azide lipids can be easily decorated with various functional groups (Oude Blenke et al., 2015).

Over the past decade, attempts have been made to promote inter-biomolecular linkages using magnetic, optical, and electrical interactions. Multivalent electrostatic interactions have also been used to modify EV surfaces. EV lipid bilayers can spontaneously fuse with other membrane structures. Taste et al. utilized electrostatic interactions to allow the fusion of cationic lipids with EVs (Piffoux et al., 2018). This approach has also been successfully employed for targeted drug delivery in biosensors and tissue engineering. Engineering EV surface membranes via external cationic lipids or polymer modifications can significantly improve their ability to fuse with cells. Functionalized modifications of these polymers can result in more efficient cell fusion (Nakase and Futaki, 2015; Tamura et al., 2017; Sawada et al., 2020). Similarly, Qi et al. developed an EV delivery system using magnetic fields and transferrin-coupled superparamagnetic nanoparticles to facilitate the effective binding of these nanoparticles to the surfaces of blood-derived EVs (Qi et al., 2016).

Compared with endogenous engineering techniques, exogenous modifications to EV surfaces involve a broad range of targeting ligands and offer greater flexibility in choosing chemical tools. Moreover, the time for engineered modifications was shortened, thus improving the effectiveness of EV treatment.

## 4 Overcoming major barriers to the clinical applications of EVs

Studies have revealed that the clearance of unmodified EVs is rapid after *in vivo* administration, especially intravenous injections. EVs that are systemically administered are rapidly cleared from the blood within 2–20 min (Wiklander et al., 2015; Morishita et al., 2017). For example, plasma-derived EVs exhibited a half-life of about 7 min in mice, whereas tumor-derived EVs were cleared from circulation more rapidly, exhibiting half-lives of around 2 min (Takahashi et al., 2013; Matsumoto et al., 2020). A pharmacokinetic study revealed that most EV doses do not reach the target tissues but accumulate nonspecifically in the liver and spleen, with a fraction directed to the kidneys, lungs, and gastrointestinal tract (Kooijmans et al., 2012; Lai et al., 2014). However, EV clearance by the spleen was reported to be the most significant (Cataldi et al., 2017).

The underlying mechanisms should be explored to develop improved versions of EVs that can escape organism capture and exhibit potent biological activities. Scientists have made significant efforts to modify EVs and demonstrated their feasibility and flexibility to undergo engineering modifications (Liang et al., 2021). In summary, the rapid clearance of EVs as therapeutic or drug delivery vehicles after administration is a limitation in certain clinical settings. To facilitate the clinical translation of EVs and broaden their application in most clinical settings, it is essential to use bioengineering technologies for the function modification of these EVs. The functionalized modifications of EVs can improve their abilities to deliver therapeutic molecules to the target site, thereby enhancing their therapeutic effects (Zhang et al., 2020a). Until now, there have been relatively few studies exploring the application potential of dental-derived EVs in the field of regenerative medicine. To expedite their development, this discussion focuses on the prospects of cutting-edge engineered EV approaches with promising applications from two perspectives: targeting efficiency and *in vivo* circulation time. Tables 3, 4 summarize the applications of engineered EVs with high targeting efficiency and long circulation time.

**TABLE 3:** Summary of EV targeting strategies used in regenerative medicine.

Targeting moieties	Target	Method of conjunction	Cargo	Loading method	Administration	EV/exosome origin	Application areas	Ref
RGD	αvβ3 overexpressing cells (HUVEC)	DSPE-PEG-RGD	Ac4ManNAz	Reversible permeabilization, click chemistry	*In vitro*/*in vivo* (zebrafish)	K562	Vascular regeneration	Wang et al. (2017)
CPP/CP05	Exosome/Dystrophin	CD63	PMO	Conjugated to CP05 via an amide linker	*In vivo* (Mouse mdx model)	Human serum	Muscle regeneration	Fletcher et al. (2007), Gao et al. (2018)
CAP	Chondrocytes	lamp2b	miR-140	Electroporation	*In vitro*/*in vivo* (rat osteoarthritis model)	Dendritic cells	Cartilage regeneration	Liang et al. (2020)
NP41/CP05	Exosome/Nerve tissue	CD63	None	None	*In vitro*	Human serum	Neurogenesis	Whitney et al. (2011), Gao et al. (2018)
CP05	Exosome/bone tissue	CD63	VEGF	Electroporation	*In vitro*/*in vivo* (radial defect rat model)	ATDC5	Bone & Vascular Regeneration	Gao et al. (2018), Zha et al. (2021)
RGE	Neuropilin-1	Click chemistry	Cur/SPION	Electroporation	*In vitro*/*In vivo* (orthotopic glioma mouse model)	Mouse macrophage cell	Tumor	Jia et al. (2018)
RVG	Neuronal cells	Lamp2b	BACE1 siRNA	Electroporation	*In vivo*	Dendritic cells	Neurogenesis	Alvarez-Erviti et al. (2011)
RVG	Cortical neural progenitors	Lamp2b	miRNA-124	Electroporation	*In vivo*	Dendritic cells	Neurogenesis	Alvarez-Erviti et al. (2011)

**Abbreviations**: Arg-Gly-Asp; RVG, rabies virus glycoprotein; Ac4ManNAz, tetra acetylated N-azidoacetyl-d-mannosamine; CP05, exosomal capture peptides; PMO, phosphorodiamidate morpholino oligomer; CPP, cell-penetrating peptide; CAP, chondrocyte-affinity peptide; NP41, NTQTLAKAPEHT; RGE, RGERPPR; VEGF, vascular endothelial growth factor; Cur, curcumin; SPION, superparamagnetic iron oxide nanoparticles.

**TABLE 4:** Methods for prolonging EV circulation time.

Modifying moieties	Engineering modification methods	EV origin	Main achievements	Ref
PEO	Chol-DNA	Mesenchymal stem cells	Higher stability and blood circulation time without altering tissue distribution profiles	Lathwal et al. (2021)
Dextran sulfate	Parental cell co-incubation	HEK293 and all mouse cell lines	Decreased EV liver clearance in mice and a significant increase in EV production	Watson et al. (2016)
cCHP nano gel	Polyvalent electrostatic interactions/Chol	Mouse macrophage cells	Efficient delivery in a functionally intact state	Sawada et al. (2020)
PEG	PEG-lipids	Neuro 2A	Allowed the application of a range of targeted ligands/antibodies	Kooijmans et al. (2016b)
			Improved cell specificity and prolonged circulation time	
ABD	Lamp2B	HEK-293T and AEC cells	Extended cycle time of EV and enrichment of EVs in lymph nodes	Liang et al. (2022)
PHA	MGE/click chemistry	MDA-MB-231 and HCT-116	The PHA-EVs exhibited high targeting efficiency with prolonged circulation in the bloodstream of animal models with tumor and RA	Lim et al. (2021)

**Abbreviations**: PEO, polyethylene oxide; PEG, polyethylene glycol; ABD, albumin binding domains; Chol, cholesteryl; PHA, PEGylated hyaluronic acid; MGE, metabolic glycoengineering; MDA-MB-231, a human breast cancer cell line; HCT-116, a human colon carcinoma cell line; HEK, human embryonic kidney cell; AEC, alveolar epithelial cell; RA, rheumatoid arthritis.

### 4.1 Targeting efficiency

Research on drug delivery using EVs as carriers has rapidly progressed. Targeting determines whether EVs can deliver drugs to diseased cells or tissues precisely and efficiently (Sharif et al., 2018; Xie et al., 2019). The regenerative potential of EVs is believed to be governed by the regulation of specific intracellular pathways. However, the use of EVs may produce serious off-target effects. Hence, it is imperative to elucidate these mechanisms and their regulation in both target and off-target tissues. To address this concern, engineering modified EVs and directing them to the therapeutic site while precisely controlling the intracellular pathways influenced by modulating the therapeutic content of EVs will significantly enhance their clinical applicability (Nagelkerke et al., 2021).

In some cases, the ability to target EVs is related to the presence of specific surface proteins. Integrins on the surface of EV are considered essential mediators of their accumulation in specific tissues (Hoshino et al., 2015). Tetratransmembrane proteins and their association with integrins have also been associated with EV targeting *in vivo*. For example, EVs containing Tspan8 were notably enriched in the pancreas of mice following intravenous injection, while co-expression of β4 integrins led to their substantial accumulation in the lungs (Rana et al., 2012). Nevertheless, the inherent tissue-specific tropism of EVs is generally limited and often insufficient for determining its maximum accumulation in organs beyond the liver and spleen. Therefore, artificial targeting modifications of EVs are required.

The surface proteins of natural EVs indeed influence their biodistribution. In accordance with this fundamental principle, surface functionalization methods, which encompass chemical treatment, affinity-based immobilization, and enzymatic ligation, bestow EVs with targeting capabilities. Receptors, antibodies, ligands, peptides, RNA adaptors, and sugar fractions can be used for EV surface functionalization (Jia et al., 2018; Pham et al., 2021).

Erviti *et al.* successfully synthesized a membrane protein fused to a neuron-specific peptide and permeated the neuron-specific rabies virus glycoprotein (RVG) peptide into EVs by transfection them to target brain cells. This was then combined with electroporation to load an exogenous siRNA on EVs. The intravenous injection of RVG-EV-siRNA delivered the siRNA specifically to the brain’s neurons, microglia, and oligodendrocytes, resulting in the knockdown of specific genes (Alvarez-Erviti et al., 2011). Jia integrated superparamagnetic iron oxide nanoparticles and curcumin into EVs using an electroporation method and further functionalized the EV surface with the peptide RGERPPR, thus targeting neuropilin-1 by click chemistry. Such EVs targeting gliomas exhibit therapeutic and imaging capabilities and cross the blood–brain barrier (BBB), contributing to the accurate identification of gliomas and the improved efficacy of drugs (Jia et al., 2018). Wang used a membrane modification strategy based on donor cell-assisted designing of peptide c (RGDyK)-modified EVs using click chemistry and dialysis methods. This strategy was utilized to introduce functional cargo, resulting in improved vascular targeting with a synergistic effect on therapeutic angiogenesis and angiogenic imaging (Wang et al., 2017). Liang used the endogenous genetic engineering approach to modify lysosome-associated membrane protein 2b (Lamp2b) on the EV surface to design EVs containing the chondrocyte-affinity peptide (CAP) and used an electroporation method to load the therapeutic drug onto miR-140. The *in vitro* results showed that the intracellular miR-140 level increased up to 7.5 times the original level. Simultaneously, precisely targeted delivery to deep cartilage chondrocytes, strictly confined to the cartilage interior, was successfully achieved, effectively alleviating osteoarthritis progression in a rat model (Liang et al., 2020).

Gao *et al.* targeted the second extracellular loop of CD63 for specific binding and obtained a peptide named CP05 in CD63-expressing cells; they anchored the EVs to other EVs derived from different cell cultures and human serum. The administration of EVs loaded with a CP05-modified amyotrophic protein resulted in an 18-fold increase in the amyotrophic protein levels in the muscles of amyotrophic-protein-deficient MDX mice. Further loading of muscle-targeting peptides significantly improved muscle functions. CP05 was also relatively stable in the blood after systemic administration (Gao et al., 2018). Similarly, Zha used CP05 to construct engineered EVs loaded with vascular endothelial growth factor and combined the EVs with 3D-printed porous bone scaffolds, which significantly increased the efficiency of the EVs and effectively promoted osteogenesis and angiogenesis in segmental bone defects. This study showed the potential of EVs for vascularized bone reconstruction (Zha et al., 2021). Ma *et al.* synthesized CP05 with the type I/III collagen-binding domain. The findings demonstrated that CP05 synthesis helped EVs promote the osteogenic differentiation of BMSCs. Subsequently, they applied a hydrogel containing the synthesized CP05 in combination with EVs to treat cranial bone defect in a rat model, significantly increasing the EVs’ retention and stability (Ma et al., 2022).

This method did not disrupt the structural features of EV surface membranes, preserved the properties and *in vivo* distribution of EVs, offered increased modifiability, and significantly improved the efficiency of targeted drug delivery utilizing EVs. Thus, combining CP05 with various therapeutic/targeted peptide drugs is a promising means for engineering EVs.

### 4.2 *In vivo* circulation time

EV targeting determines the effective concentration of therapeutic agents in the targeted tissues. However, the EVs delivered to the targeted site are rapidly cleared by monocytes/macrophages or the reticuloendothelial system, resulting in reduced accumulation in the target site. Monocyte/macrophage depletion can considerably prolong the whole-body half-life of EVs in mice, suggesting that uptake by monocytes/macrophages is an important *in vivo* mechanism for clearing EVs (Bala et al., 2015; Imai et al., 2015; Morishita et al., 2015). Phagocytosis is regarded as one of the primary mechanisms of EV internalization (Hwang et al., 2015). Several engineering strategies corresponding to the mechanism of EV degradation have emerged. Macrophages recognize and phagocytose apoptotic cells by identifying phosphatidylserine (PS), a phospholipid exposed to the plasma membrane (Segawa and Nagata, 2015; Matsumura et al., 2019). As most EVs display PS on their surface (Subra et al., 2007), they are rapidly excluded from the bloodstream. The C1C2 structural domain of a PS-binding protein can mask the PS of EVs using cadherin to prolong their circulating half-life (Kooijmans et al., 2018; Longatti et al., 2018). CD47 displayed on the surface protects EVs from phagocytosis by monocytes and macrophages, thus enhancing EV retention in circulation (Belhadj et al., 2020). Importantly, macrophages may phagocytose normal erythrocytes if CD47 on the EV surface occupies the signal regulatory protein alpha site, which is typically utilized to prevent phagocytosis (Buatois et al., 2018).

Watson identified the Scavenger Receptor Class A family (SR-A) as a novel receptor for EV uptake by monocytes/macrophages. *In vivo*, the blockade of SR-A by loading dextran sulfate on EVs using the parental cell co-incubation method remarkably decreased EV clearance in hepatic tissues along with increased accumulation in tumors (Watson et al., 2016). Polymers such as polyethylene glycol (PEG) or gels to encapsulate EVs can affect their pharmacokinetics and biodistribution by improving their surface physicochemical properties (Antes et al., 2018; Choi et al., 2019). Sawad synthesized engineered EVs owing to the electrostatic interactions between amphiphilic cationic cholesteryl pullulan (cCHP) nanogel polymers and anionic EV surface membranes, which significantly improved EV circulation time and enhanced their efficient delivery *in vivo* (Sawada et al., 2020). Kooijmans developed PEG-coated EVs to avoid phagocytosis by monocytes, which increased the blood circulation time of EVs from 10 min to more than 60 min (Kooijmans S.a. A. et al., 2016; Suk et al., 2016). Choi discovered that surface modification of EVs with PEG-derived 1,2-dioctadecanoyl-sn-glycero-3-phosphoethanolamine could prolong the circulation time of EVs in a similar manner (Choi et al., 2019). These studies indicate the potential of hybrid EV nanocarriers with different biocompatibilities to transport various contents *in vivo* and ensure their effective delivery in a functional state. This provides robust support for the utilization of EV-based therapies in the fields of nanomedicine and tissue engineering.

However, the toxicity and immunogenicity of PEG carrier systems should be investigated (Shiraishi and Yokoyama, 2019). Moreover, the mentioned surface encapsulation approach may mask the natural targeting or therapeutic properties of EVs. It may not control the length and number of conjugated polymers, which can be a barrier to their translation to clinical practice. To overcome these limitations, Lathwal *et al.* created a polymer-based engineered modification platform capable of precisely controlling polymer composition, chain length, and loading, which used DNA linkers to functionalize EVs. This platform was combined with atom transfer radical polymerization technology (Baker et al., 2019; Yerneni et al., 2019) to design EV–polymer hybrids (EPHs) with considerably high stability and better pharmacokinetics. Furthermore, the EV surfaces were precisely designed by using different synthetic polymers. These polymers could be easily tuned to overcome stability- and activity-related limitations *in vitro* and *in vivo*, and the circulation time of all EPH samples was prolonged. These properties of EPH overcame some major limitations associated with the clinical application of EV-based therapies (Lathwal et al., 2021). A recent research revealed that the surface of the albumin-binding domain (ABD) was displayed on the EV surface. ABDs were either present in the extracellular loops of the selected EV-rich tetra-transmembrane proteins (CD63, CD9, and CD81), or they were directly fused to the extracellular terminus of a single transmembrane EV-sorting domain (e.g., Lamp2B). These engineered EVs exhibited the ability to bind to human serum albumin both *in vitro* and *in vivo*. Experimental data showed that the circulation time of EVs increased significantly after they were injected via different routes in different strains of mice (Liang et al., 2022).

Thus, when EVs bind to tissue-specific targeting molecules, the circulation time of EVs *in vivo* is equally critical. The combined effect of the two may markedly accelerate the translation of EV-based therapies to clinical practice.

## 5 Discussion and perspectives

EVs possess unique natural targeting capabilities, have the ability to cross the BBB, and display low immunogenicity, enabling effective intercellular communication and the delivery of molecules to distant sites. These characteristics provide significant advantages over existing drug delivery vehicles. To fully exploit their potential and benefits and develop efficient and reproducible nanocarrier drug delivery systems, functionalized modifications of EVs combined with emerging bioengineering technologies are necessary to adapt them to most clinical therapeutic settings. The endogenous genetic modification of EV parental cells or the exogenous direct functionalization of isolated EV surfaces can help achieve this adaptation. Both approaches have advantages and should be selected according to specific application conditions. Notably, DSC-EVs exert a natural therapeutic effect and can be used to enhance the therapeutic effect of loaded drugs. Enhanced knowledge of the molecular content and biological functions of EVs has paved the way for the development of various engineering approaches aimed at improving the therapeutic or targeting efficacy of EVs at the preclinical stage. In addition, a more detailed understanding of specific markers and functions associated with EVs derived from different parental cells can significantly assist in establishing criteria for using optimal EV populations with maximum efficiency in therapeutic and diagnostic applications. Consequently, when selecting and designing target EVs, the natural targeting properties of different EVs should be considered because their specific target effects can more easily be further amplified by engineering modifications.

However, owing to the complexity of EV biogenesis and the wide range of engineered modification strategies, identifying a generic strategy for precisely modifying biotherapeutic cargo in EVs is challenging. Enhanced comprehension of EV biogenesis, surface markers, cargo, uptake, and function enables accurate discrimination among distinct EV types and facilitates the development of treatment schemes without adverse effects. Various engineering approaches are available to add desired functionality to EVs. Exogenous reagents can be introduced into EVs before or after vesicle formation. Genetic and chemical engineering of cells can manipulate EV cargo. Post-formation loading techniques encompass mixing, electroporation, sonication, hypotonic dialysis, extrusion, freeze-thaw cycles, and the utilization of saponin or commercially available transfection reagents. Furthermore, surface modifications of EVs can facilitate tissue-specific therapeutic delivery (Bahmani and Ullah, 2022). Hence, it is crucial to carefully choose modification techniques while also gaining a deeper comprehension of EVs. The utilization of the known molecular mechanisms associated with EVs from particular sources to guide the selection of suitable modification methods is paramount for the success of engineered EV applications.

Despite extensive endeavors to develop diverse engineering techniques for the functionalization of natural EVs and promising preclinical outcomes in regenerative medicine applications, the clinical translation of natural EVs continues to pose significant challenges. Firstly, transplantation protocols for human cells and tissues involve many ethical and policy issues related to informed consent, quality, procurement safety, processing of biological tissues, and distribution and international circulation of human cells and tissues. Maintaining regulatory order requires the coordinated operation of multiple parties. Secondly, EVs lack the capability to respond to pathological environmental stimuli as effectively as stem cells. It is important to note that clinical studies on stem cells cannot be directly transplanted to stem cell-secreted EVs because the therapeutic mechanisms of EVs may diverge significantly from those of their stem cell source. Consequently, gaining a profound understanding of EV biology and its unique action mechanisms in specific diseases is a daunting task for researchers. Furthermore, the lack of adequate and reproducible production methods remains a significant limitation to this day, and the future of EV therapy depends on the realization of high-purity isolation and mass production of EVs. Therefore, there is an urgent need to develop improved technologies for vesicle isolation, purification, characterization, and storage. In addition, such technologies should be clinically scalable to enable rapid translation of promising EV-based therapies. Despite the daunting challenges, with all the efforts to establish order in the regulatory system of stem cell banking at the local and national levels, the deepening knowledge of EV biology and therapeutic mechanisms, and the solid multidisciplinary collaborations in the fields of bioengineering, chemistry, materials science, nanotechnology, clinical medicine, and industry, these emerging positives will surely propel safe and effective EV nanotherapies toward clinical translation.

Manufactured EVs have been proposed as an alternative to natural EVs in optimizing EV therapeutics and clinical translation. Manufactured EVs, also known as synthetic lipid nanoparticles (LNPs), offer advantages over natural EVs in yield and cost-effectiveness. Synthetic LNPs are more cost-effective and accessible to manufacture on a large scale than EVs. The potential benefits of EVs over synthetic LNPs stem from their intrinsic targeting properties and immune-interacting proteins on the surface of EVs. EV is often described as having an intrinsic tissue-targeting ability when administered *in vivo*. EV from specific cancer cell lines has been shown to accumulate in specific organs after *in vivo* injection (Hoshino et al., 2015). Engineered EVs, in particular, are considered safer delivery vehicles for RNA or gene editing tools than other synthetic nanoparticles (Maugeri et al., 2019). However, any isoform protein is a potential antigen and immune target. Therefore, synthetic LNPs lacking homodimeric proteins may be advantageous in avoiding premature recognition and destruction (Chen W. C. et al., 2013). Furthermore, the growing interest in isolating EVs from different sources opens up the possibility of optimizing EV-based therapies for each specific clinical purpose. EVS released from various cell lines possess distinct properties. Obtaining well-known sources of MSCs, such as bone marrow and adipose tissue, can often be challenging. Therefore, the exploration of new alternative sources of highly plastic and accessible stem cell EVs has the potential to optimize EV therapies. DSCs have a higher proliferative capacity compared to the most widely studied BMSCs. DSCs are more acceptable to patients in terms of accessibility and prognosis. DSCs are more pleasing to patients than BMSCs and, in most cases, are benign reuse of discarded oral biologic tissues.

Furthermore, while current foundational research on DSCs tends to focus on dental applications, a significant body of research suggests that the potential of DSCs and their derived EVs extends beyond the dental field. Their therapeutic potential has been explored in diverse areas, such as the treatment of spinal cord injuries, brain injuries, myocardial infarction, wound healing, and bone replacement (Cordeiro et al., 2008). Given their self-renewal ability and strong plasticity, these cells can encompass various clinical applications in medicine and dentistry. However, it is worth noting that DSC has apparent drawbacks; one remarkable concern is whether the final amount of EV isolated from stem cells obtained from a single tooth after a normal expansion period would be sufficient to effectively treat a specific disease with EV therapy. This limitation arises because not all people have wasted wisdom teeth, and the availability of DSCs from deciduous teeth is much more limited. Consequently, this leads to very limited clinical scenarios. In this regard, establishing stem cell banks and maintaining order, as discussed earlier, are particularly critical. In addition, mature DSC acquisition and preservation programs, subsequent DSC *in vitro* expansion programs, and enrichment of derived EV biology are also essential.

## 6 Conclusion

EVs have great potential to become therapeutic targets, biomarkers, innovative drug delivery systems, and stand-alone therapeutic agents. However, nanotherapies utilizing EVs as regenerative medicine are still in their infancy. These vesicles play a crucial role in regulatory or pathological intercellular communication, and their circulation in the body influences their biodistribution and the effective EV dose reaching the target tissue.

In regenerative medicine, it is advisable to choose parental stem cells characterized by high plasticity and proliferative capacity. Additionally, employing emerging bioengineering techniques can enhance the efficiency and *in vivo* circulation duration of EVs, facilitating the delivery of therapeutic doses to specific tissues.

With the synergistic development of all related industries, the clinical translatability of EV therapies will be accelerated in the future. Overall, this study summarized the research progress of dental stem cell-derived EVs in oral tissue regeneration and discussed the current strategies for loading and modifying the functional cargo of engineered EVs. Furthermore, it has also addressed the progress and prospects of engineered EVs in tissue engineering and regenerative medicine. The primary goal of this study is to provide a strong basis for developing EV-based strategies for clinical applications.

## Figures and Tables

**Table:** 

**Extracellular vesicle (EV)**	membranous, lipid-bilayer enclosed vesicle released by cells that may contain full-length transmembrane proteins, soluble proteins in the vesicular lumen, as well as RNA and DNA, depending on vesicle type.
**Mesenchymal stem cells (MSCs)**	multipotent stromal cells that can differentiate into a variety of cell types, including osteoblasts (bone cells), chondrocytes (cartilage cells), myocytes (muscle cells) and adipocytes (fat cells which give rise to marrow adipose tissue).
**Human bone marrow (hBM)**	a semi-solid tissue found within the cancellous portions of bones. It is composed of hematopoietic cells, marrow adipose tissue, and supportive stromal cells.
**Dental pulp stem cells (DPSCs)**	DPSCs are a mesenchymal type of stem cells inside dental pulp. DPSCs have osteogenic and chondrogenic potential in vitro and can differentiate into dentin, in vivo and also differentiate into dentin-pulp-like complex.
**Icrovesicle**	type of small to large 150–1000-nm ectosome derived by direct outward budding of the plasma membrane followed by pinching off of the vesicle.
**Stem cells from exfoliated deciduous teeth (SHEDs)**	exfoliated human deciduous tooth contains multipotent stem cells.
**Periodontal ligament stem cells (PDLSCs)**	It is an adult stem cell derived from periodontal ligament tissue and has the basic characteristics of an adult stem cell.
**Stem cells from apical papilla (SCAPs)**	residing in the apical papilla of immature permanent teeth represent a novel population of dental MSCs that possess the properties of high proliferative potential, the self-renewal ability, and low immunogenicity.
**Gingival MSCs (GMSCs)**	It is an undifferentiated cell present in human gingival mesenchymal tissue with the ability of self-renewal, multidirectional differentiation and immune regulation.
**Dentin sialophosphoprotein (DSPP)**	It is a non-collagenous protein of dentin, mainly expressed by dentin cells, and is currently recognized as a tooth-specific protein.
**Dentin matrix acidic phosphoprotein 1 (DMP1)**	It is an extracellular matrix protein and a member of the small integrin-binding ligand N-linked glycoprotein family.
**miRNA**	small noncoding RNA that exerts post-transcriptional regulation of gene expression through RNA silencing.
**Glycosylphosphatidylinositol (GPI)**	a phosphoglyceride that can be attached to the C-terminus of a protein during posttranslational modification. The resulting GPI-anchored proteins play key roles in a wide variety of biological processes.
**Indocyanine green(ICG)**	a medical dye used in tests to help determine the cardiac (heart) output, liver function, and blood flow in the liver.
**Azide (N3)**	Azide anion is a pseudohalide anion. It has a role as a mitochondrial respiratory-chain inhibitor. It is a conjugate base of a hydrogen azide.
**Rabies virus glycoprotein (RVG)**	It is the only structural protein exposed outside the rabies virus membrane.
**Blood–brain barrier (BBB)**	It refers to the barriers between plasma and brain cells formed by the walls of brain capillaries and neuroglia and between plasma and cerebrospinal fluid formed by the choroid plexus, which prevent certain substances, mostly harmful, from entering the brain tissue from the bloodstream.
**Lysosome-associated membrane protein 2b (Lamp2b)**	It is a member of the lysosome-associated membrane protein (LAMP) family, which is mainly localized in lysosomes and endosomes, with a small amount on the cell surface, and is also abundantly expressed on the surface of exosomes.
**Chondrocyte-affinity peptide (CAP)**	It belongs to a kind of bioactive peptide, which consists of specific amino acid sequences, and can be used for affinity effect on some key factors of cartilage tissue engineering through identification and screening to improve the effectiveness of cartilage tissue engineering repair.
**Phosphatidylserine (PS)**	It is a component of the cell membrane of neuronal cells and activates protein kinase C .
**Scavenger Receptor Class A family (SR-A)**	Based on the structure and origin of scavenger receptor molecules, they have been categorized into eight subfamilies from A-H, with class A scavenger receptors being one of these classes.
**Polyethylene glycol (PEG)**	It is a polymerization of polyethylene oxide and water.
**Exosome(EXO)**	type of small 30–150-nm EV derived from the release of ILVs upon fusion of an MVE or amphisome with the plasma membrane.
**Intraluminal vesicle (ILV)**	small vesicle that buds from the limiting membrane into the endosomal lumen of developing MVEs.
**Apoptotic extracellular vesicle**	class of EVs ranging in size from 200 to 1000 nm apoptotic vesicles to 1 to 5-μm apoptotic bodies; generated when cells undergo programmed cell death (apoptosis).
**Major histocompatibility complex (MHC)**	group of genes that code for proteins found on the surfaces of cells that help the immune system recognize foreign substances.
**single-chain Fragment Variable (scFV)**	small-sized artificial constructs composed of the immunoglobulin heavy and light chain variable regions connected by a peptide linker
